# Intussusception after Rotavirus Vaccine Introduction in India

**DOI:** 10.1056/NEJMoa2002276

**Published:** 2020-11-12

**Authors:** Samarasimha Nusi Reddy, Nayana Prabhakaran Nair, Jacqueline Elizabeth Tate, Varunkumar Thiyagarajan, Sidhartha Giri, Ira Paraharaj, Venkata Raghava Mohan, Sudhir Babji, Mohan Digambar Gupte, Rashmi Arora, Sunita Bidari, Sowmiya Senthamizh, Suhasini Mekala, Krishna Babu Goru, Bhaskar Reddy, Padmalatha Pamu, Rajendra Prasad Gorthi, Manohar Badur, Vittal Mohan, Saroj Sathpathy, Hiranya Mohanty, Mrutunjay Dash, Nirmal Kumar Mohakud, Rajib Kumar Ray, Prasantajyoti Mohanty, Geetha Gathwala, Suraj Chawla, Madhu Gupta, Rajkumar Gupta, Suresh Goyal, Pramod Sharma, Mannancheril Abraham Mathew, Tarun John Kochukaleekal Jacob, Balasubramaniyan Sundaram, Girish Kumar Chethrapilly Purusothaman, Priyadarishini Dorairaj, Muthukumaran Jagannatham, Kulandaivel Murugiah, Hemanthkumar Boopathy, Raghul Maniam, Rajamani Gurusamy, Sambandan Kumaravel, Ashwitha Shenoy, Hemant Jain, Jayanta Kumar Goswami, Ashish Wakhlu, Vineeta Gupta, Gopinath Vinayagamurthy, Umesh D. Parashar, Gagandeep Kang

**Affiliations:** 1 The Wellcome Trust Research Laboratory, Division of Gastrointestinal Sciences, Christian Medical College, Vellore, Tamil Nadu, India.; 2 Centers for Disease Control and Prevention, Atlanta, GA, USA; 3 Indian Council of Medical Research, New Delhi, India; 4 Department of Community Health, Christian Medical College, Vellore, Tamil Nadu, India; 5 Translational Health Science and Technology Institute, Faridabad, India; 6 Kurnool Medical College and Government General Hospital, Kurnool, Andhra Pradesh, India; 7 Government General Hospital and Rangaraya Medical College, Kakinada, Andhra Pradesh, India; 8 King George Hospital and Andhra Medical College, Visakhapatnam, Andhra Pradesh, India; 9 Sri Venkateshwara Medical College, Tirupati, Andhra Pradesh, India; 10 Sardar Valla Bhai Patel Post Graduate Institute of Paediatrics, Cuttack, Odisha, India; 11 Institute of Medical Sciences and SUM Hospital, Bhubaneswar, Odisha, India; 12 Kalinga Institute of Medical Sciences, Bhubaneswar, Odisha, India; 13 Hi-Tech Hospital, Bhubaneswar, Odisha, India; 14 Pandit Bhagwat Dayal Sharma Post Graduate Institute of Medical Sciences, Rohtak, Haryana, India; 15 Shaheed Hasan Khan Mewati Government Medical College, Mewat, Haryana, India; 16 Post Graduate Institute of Medical Education and Research, Chandigarh, India; 17 Sawai Man Singh Medical College, Jaipur, Rajasthan, India; 18 Rabindranath Tagore Medical College, Udaipur, Rajasthan, India; 19 Dr. Sampurnanand Medical College, Jodhpur, Rajasthan, India,; 20 Malankara Orthodox Syrian Church Medical College Hospital, Kolencherry, Kerala, India; 21 Christian Medical College, Vellore, Tamil Nadu, India; 22 Kanchi Kamakoti Child Trust Hospital, Chennai, Tamil Nadu, India; 23 National Institute of Epidemiology, Chennai, Tamil Nadu, India; 24 Institute of Child Health, Chennai, Tamil Nadu, India; 25 Government Rajaji Hospital and Madurai Medical College, Madurai, Tamil Nadu, India; 26 Coimbatore Medical College, Coimbatore, Tamil Nadu,; 27 Jawaharlal Nehru Institute of Post-graduate Medical Education & Research, Puducherry, India; 28 Mahatma Gandhi Memorial Medical College, Indore, Madhya Pradesh, India; 29 Government Medical College, Guwahati, Assam, India; 30 King George Medical College, Lucknow, Uttar Pradesh, India; 31 Institute of Medical Sciences, Banaras Hindu University, Varanasi, Uttar Pradesh, India; 32 Government Vellore Medical College, Vellore. Tamil Nadu, India

**Keywords:** Intussusception, India, Infants, Safety, Rotavirus, Vaccines

## Abstract

**Background:**

Rotavac®, an Indian-made, 3-dose, oral rotavirus vaccine, was introduced in the universal immunization program in India in 2016. Pre-licensure safety data for the vaccine were limited to a single trial of 6800 Indian infants; here we report results of a post-marketing surveillance study to assess a level of intussusception risk similar to that seen with other multinational rotavirus vaccines in other countries.

**Methods:**

Multicentric hospital-based active surveillance was conducted at 27 Indian hospitals from April 2016 to June 2019. Children meeting Brighton level 1 criteria of radiological or surgical confirmation of intussusception were enrolled and vaccination was ascertained through vaccination records. The relative incidence (RI) for intussusception within 1-7, 8-21 and 1-21-days post-vaccination in children 28-365 days of age was evaluated by self-controlled case-series (SCCS) analysis. For a subset, a matched case-control analysis was performed with age-, gender- and location-matched controls.

**Results:**

970 cases were enrolled, and 589 children 28-365 days of age were included in the SCCS analysis. Post-dose 1, intussusception relative incidence (RI) was 0.83 (95% CI 0.0, 3.00) and 0.35 (95% CI 0.0, 1.09) in the 1-7 and 8-21 day windows, respectively. Similar results were observed post-dose 2 (RI=0.86 (95% CI 0.20, 2.15) and 1.23 (95% CI 0.60, 2.10), respectively), and post-dose 3 (RI=1.65 (95% CI 0.82, 2.64) and 1.08 (95% CI 0.69, 1.73), respectively). No increase in intussusception risk was found in the case-control analysis.

**Conclusion:**

The rotavirus vaccine produced in India and evaluated here was not associated with intussusception in Indian infants.

Post-licensure studies with rotavirus vaccines have demonstrated varying risk of intussusception in different settings worldwide. The association of intussusception with rotavirus vaccination was identified in 1998, when RotaShield® (Wyeth Lederle Vaccines, USA), the first licensed rotavirus vaccine, was withdrawn because of an increased risk of intussusception^1,2^. Subsequent large pre-licensure trials of the second-generation rotavirus vaccines Rotarix® (GlaxoSmithKline Biologicals, Rixensart, Belgium) and RotaTeq® (Merck & Co. Inc.,USA) did not identify increased risk of intussusception in clinical trials with 65,000-70,000 infants ^3,4^. However, postmarketing surveillance for Rotarix®-- in Mexico, Brazil, USA, Australia and England found 1-6 excess cases of intussusception per 100,000 vaccinated children^5-10^. Post-marketing surveillance for RotaTeq® in the USA and Australia found 1-7 excess cases per 100,000 vaccinated children^6,10^. Despite the hypothesis that intussusception might be an adverse event associated with all rotavirus vaccines^11^, the World Health Organization (WHO) recommended rotavirus vaccine introduction into childhood vaccination programs as cases and deaths averted due to diarrhea are greater than the additional intussusception, resulting in a favourable risk benefit analysis^12^. Recently, our understanding of the safety of rotavirus vaccination in specific populations was further informed by the finding that in seven low-income African countries and South Africa, where vaccine efficacy has been lower than that seen in high-income countries, there was no increased risk of intussusception following Rotarix® vaccination^13,14^.

The vaccine studied here, Rotavac® (Bharat Biotech International Ltd, Hyderabad, India), is an oral monovalent, live attenuated rotavirus vaccine containing a naturally occurring bovine-human reassortant 116E strain (G9P[11])^15,16^. This vaccine is given as a 3-dose series at 6, 10, and 14 weeks of age, concurrent with other childhood vaccines. It had an efficacy of 56% against severe rotavirus gastroenteritis in a multi-site Indian phase 3 clinical trial and was licensed in 2014^17^. The trial, in 6799 infants randomized 2:1 to vaccine and placebo, was not large enough to detect a small increased risk of intussusception^17^. This vaccine was introduced into the Universal Immunization Programme of India^18^ in four states in 2016, five in 2017, one in 2018 and 10 additional states in 2019^19^. More than 100 million doses have been administered to Indian infants.

There are limited background data on intussusception in India. Two studies have reported a general incidence of intussusception of 18/100,000 infants and 20/100,000 infants^20,21^. The Indian National Technical Advisory Group on Immunization and the WHO recommended monitoring of vaccine safety after introduction into the immunization program^22^, in response to which we established the Indian Intussusception Surveillance Network^23^. Since the vaccine on which we now report is WHO pre-qualified, safety data are important for India, for the Gavi Alliance and for countries considering the introduction of rotavirus vaccines.

## Methods

### Study Sites

Active intussusception surveillance was conducted at 27 participating hospitals (Supplementary Appendix, Table S1) that could carry out sentinel surveillance (here termed sentinel hospitals) in ten Indian states in which half the population of India resides. Surveillance started in four states in April 2016 and was expanded concurrently with vaccine introduction. The protocol, previously published,^23^ has detailed methods and is also posted at **NEJM.org**. All children less than two years of age and meeting level 1 diagnostic certainty for intussusception per Brighton collaboration criteria were eligible for recruitment. Level 1 Brighton collaboration criteria require the confirmation of intussusception by radiologic findings (specifically, if reduced by pneumatic/hydrostatic/contrast enema), and/or during surgery or at autopsy (Table S2)^24^.

Surveillance staff completed paper case report forms (CRFs) with socio-demographic and clinical details, treatment and outcomes, and obtained copies of ultrasound images and reports, and treatment notes. From the parents/guardian, information on rotavirus vaccination status and a copy of the vaccination record were collected and dates of first, second and third vaccination recorded. For unvaccinated and partially vaccinated children, the child’s health sub-center/primary health center were contacted to verify vaccination status. For a subset of 162 enrolled cases, we enrolled an age- (date of birth±30 days), gender, and location-matched (same state of residence) control who was admitted with illness unrelated to the gastrointestinal tract within 30 days of the admission of the case. Vaccination card copies and information were collected as for cases. All CRFs were sent to the central data management team at Christian Medical College, Vellore and entered into an audit trial enabled SQL database, where data cleaning and query resolution from sites were managed and validated against documents for 10% of all CRFs. This study was approved by the institutional review board of Christian Medical College, Vellore and institutional ethical committees of all participating hospitals. Written informed consent was obtained from the parents/guardians of all enrolled cases and controls.

### Statistical Analysis

#### Self-Controlled Case Series Analysis

To detect a relative incidence (RI) of 2, with a 21-day risk period after any dose, with 80% power and 5% level of significance, we required 160 cases^25^, but for a RI of 2 after the first dose the sample size was 263 cases ^25^. The self-controlled case-series (SCCS) method was used to assess the intussusception risk after vaccine administration. The relative incidences (RIs) were calculated using conditional Poisson regression analysis by comparing the incidence in the risk period i.e 1-7 days, 8-21 days, 1-21 days after each dose of vaccine with the incidence in all other observational periods (non–risk periods) for each case as required for SCCS analysis^23,26,27^. The pseudo-likelihood method^27^ was used to allow the contraindication of vaccination after an episode of intussusception and event ascertainment was independent of vaccination status. The analysis was restricted to children aged 28-365 days at the time of symptom onset considering the minimum and maximum ages at which vaccination was given. Children with a recurrent episode of intussusception were excluded. Children with verified vaccination history were included in SCCS analysis, and children in whom vaccination history was only based on parental reports or who had received a different rotavirus vaccine were excluded. Unvaccinated children were included in the analysis to adjust for the background incidence of intussusception by age. Age was controlled in the model using 14-day window periods. The confidence interval estimates were derived by bootstrapping with 1000 iterations. For all children, we attempted follow up at approximately 18 months of age. During follow-up, data were collected about the vital status of the child (alive/dead), repeated intussusception and receipt of additional doses of rotavirus vaccine after the intussusception.

#### Matched Case-control Analysis

The matched case-control analysis was conducted on a subset of intussusception cases from the SCCS analysis for which matched controls were enrolled. Rotavirus vaccination status with confirmed vaccination was needed for both the case and matched control for the pair to be included. Conditional logistic regression was used to assess the ratio of odds that cases and age-, gender- and location-matched controls were vaccinated in the same risk window. A reference date was created for controls, which was the date on which control was the same age as their respective case at the time of symptom onset. Exposure to the vaccine with the first, second or third dose in the risk windows of 1-7, 8-21 and 1-21 days prior to reference date was determined. The matched odds ratios are reported as point estimates with 95% confidence intervals. Sensitivity analyses for both the SCCS and matched case-control analyses used date of admission instead of date of symptom onset. All statistical analyses were performed using STATA version 13.1.

GK, JET, UDP designed the study, SR, NPN led the data acquisition with all investigators and wrote the first draft, JET, SR, NPN and VT analysed the data, GK vouches for the data, analysis and decision to publish.

## Results

970 children <2 years of age with intussusception meeting the Brighton level 1 case definition were enrolled (Table S1). Of these, 258 children were excluded from the analysis as they were aged less than 28 days or more than 365 days. Of 712 children aged 28-365 days, 46 children did not have vaccination card copies, 40 children had received a vaccine other than the one under study; the rotavirus vaccination status could not be verified by the health sub-center/primary health center for 37 children. Thus, 589 children were included in the SCCS analysis (Supplementary Appendix, Fig. S1).

### Patient Characteristics and Clinical Features

Of the 589 intussusception cases included in the SCCS analysis, the median (IQR) age was 7 (5-9) months (Table S3). Intussusception was more common among male patients with a male: female ratio of 2:1. Blood in stools and vomiting were the most common symptoms --- 481 (82%) for blood in the stool and 438 (74%) for vomiting. Other than constipation and blood in stools, there were no significant differences in vaccinated and unvaccinated children (Table S4). Ileo-colic intussusception was most common, seen in 498 (84%) followed by ileo-ileal in 33 (6%) children. The treatment modalities were hydrostatic/pneumatic reduction (200, 34%) surgical reduction (321,54%) and intestinal resection (68, 12%). There were 6 deaths with a case fatality rate of 1% (Table S3).

### Vaccine Coverage and Vaccination Timing

**Figure 1:**
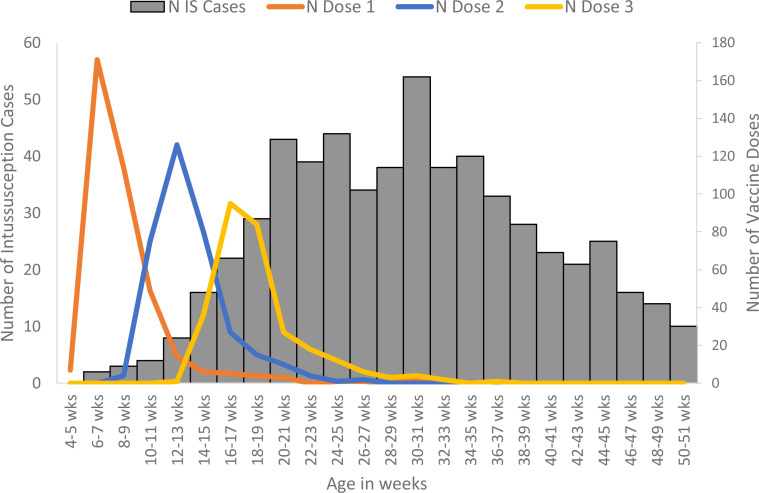
Age at immunization and at onset of intussusception (IS) in Indian infants included in the SCCS analysis from 27 hospitals in ten Indian states, April 2016 through June 2019

Among 589 children, 289 (49%) children had received all three doses, 55 (9%) two doses, 33 (6%) one dose, and 212 (36%) did not receive any dose. The median age (IQR) at first, second and third doses were 8 (7-9), 13 (12-14), and 18 (16-20) weeks, respectively. Of the 377 children who received the first dose of rotavirus vaccine, 330 (87.5%) children received oral polio vaccine on the same day. Of the 344 and 289 children who received second and third doses of rotavirus vaccine, 300 (87.2%) and 240 (83%) of such children received second and third dose of oral polio vaccine on the same day. The third dose of vaccine is scheduled at 14 weeks, but children presented at a median age of 18 weeks, which overlapped with the peak age of intussusception (Fig. 1).

### Follow-up of Children in the SCCS Analysis

We were able to recontact 455/589 children at a median (IQR) age of 16 (13-22) months. Of those, 8/455 (1.8%) had a repeat episode of intussusception and 7 (1.5%) died after hospital discharge with deaths occurring between 4 and 15 months after discharge and none due to intussusception. Even though further doses of the vaccine were contraindicated after an intussusception by the manufacturer, parents reported that 22 (7.3%) of 300 children who had not completed their rotavirus immunization series had received at least one dose of rotavirus vaccine after intussusception (Table S5).

### Risk of Intussusception after Vaccination

#### Self-Controlled Case-Series Analysis

**Figure 2:**
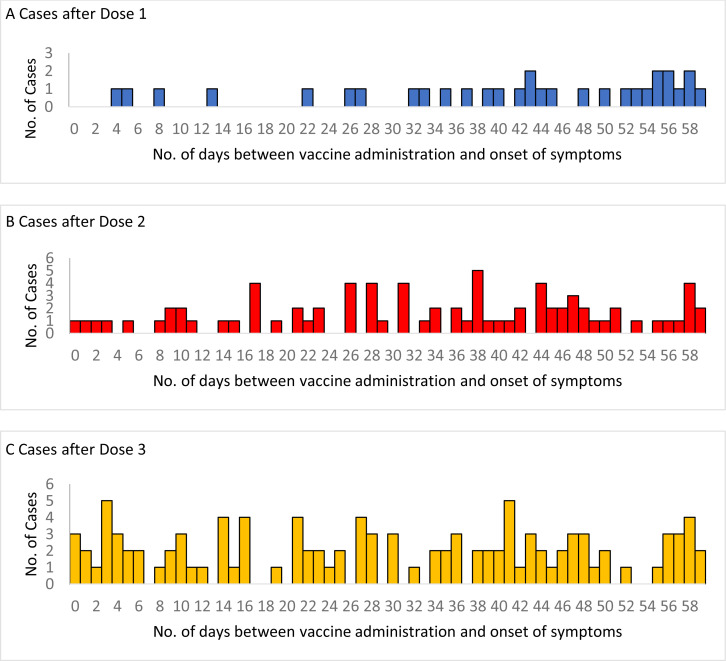
Cases of intussusception occurring in the 0-59 days# after dose 1, dose 2 and dose 3 of Rotavac® vaccine from 27 hospitals in 10 Indian states, April 2016 through June 2019 # An additional 345 cases occurred more than 60 days after dose 1, an additional 265 cases occurred more than 60 days after dose 2, and an additional 181 cases occurred more than 60 days after dose 3

After dose 1, 2 cases occurred in the risk period of 1-7 day sand 2 cases in the 8-21 days risk period. After dose 2, 4 cases occurred in the 1-7 day and 15 cases in the 8-21 day risk periods. After dose 3, 15 and 22 cases occurred in the 1-7 day and 8-21-day risk periods, respectively (Fig. 2). The risk of intussusception in the 1-7 days (RI 0.83, 95% CI 0, 3.00) and 8-21 days (RI 0.35, 95% CI 0-1.09) after dose 1 was not higher than the background risk. The risk of intussusception in the 1-7 days and 8-21 days after dose 2 and dose 3, and for 1-21 days after any dose were also not higher than the background risk (Table 1).

**Table 1:** Relative incidence of intussusception in the risk periods after first, second and third doses of Rotavac® vaccine in age-eligible Indian infants (n=589) between 28-365 days of age with a confirmed history of having received or not received rotavirus vaccination by the self-controlled case series method.

Doses of rotavirus vaccine	Risk Period (days)	No. of cases in risk period	RI (95% CI)
**Dose 1**	1-7 days	2	0.83 (0.0-3.00)
	8-21 days	2	0.35 (0.0-1.09)
	1-21 days	4	0.52 (0.08-1.27)
**Dose 2**	1-7 days	4	0.86 (0.20-2.15)
	8-21 days	15	1.23 (0.60-2.10)
	1-21 days	19	1.13 (0.61-1.94)
**Dose 3**	1-7 days	15	1.65 (0.82-2.64)
	8-21 days	22	1.08 (0.69-1.73)
	1-21 days	37	1.24 (0.81-1.82)

# The date of intussusception was considered as the date of onset of symptoms

* Of 589 children included in the analysis, 377 (64%) were vaccinated with 1 or more dose and 212 (36%) did not receive any dose of the rotavirus vaccine under study.

**Figure:**
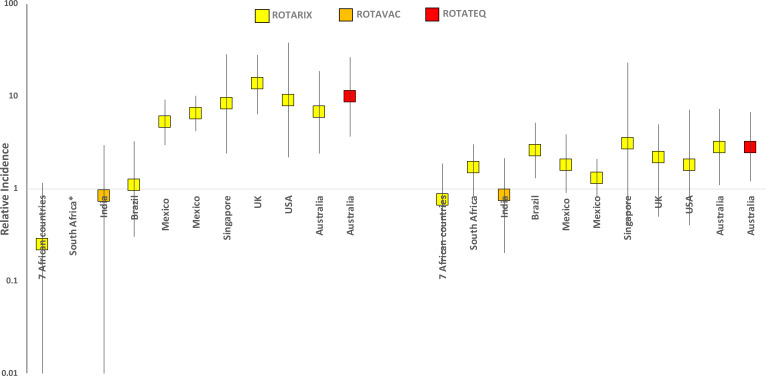


#### Matched Case-Control Analysis

For the case-control analysis, 162 intussusception cases with age-, gender- and location-matched controls with recorded vaccination history were included (Fig S2). The odds of intussusception in the 1-7 day (matched odds ratio [OR] 1.00, 95% CI 0.12-78.49) and 8-21 day (matched OR 0, 95% CI 0-1.51) risk periods after dose 1 were not significantly different in cases and controls (Table 3). Similarly, the odds of intussusception in the 1-7 days or 8-21 days after dose 2 and dose 3, or for 1-21 days after any dose were not different in cases and controls (Table 2). Odds ratios were not significantly different in all risk windows using date of admission instead of date of symptom onset for both the SCCS analysis and the matched case-control analysis (Table S6 and S7). Similar risk estimates were also obtained with the SCCS analysis restricted to include only the 162 intussusception cases that were included in the matched case-control analysis (Table S8).

**Table 2:** Matched odds of intussusception in the risk window after first, second and third dose of rotavirus vaccination in age-, gender- and location matched case-control pairs (n=162) of Indian infants with a confirmed rotavirus vaccination history with the vaccine under study

Doses of rotavirus vaccine	Risk window relative to reference date#	No. of cases in risk window	No. of controls in risk window	Matched odds ratio
Dose 1	1-7 days	1	1	1 (0.12, 78.49)
	8-21 days	1	5	0 (0, 1.51)
	1-21 days	2	6	0 (0, 1.51)
Dose 2	1-7 days	1	1	1 (0.01, 78.49)
	8-21 days	3	3	1 (0.07, 13.79)
	1-21 days	4	4	1 (0.13, 7.46)
Dose 3	1-7 days	6	3	2.5 (0.41, 26.25)
	8-21 days	7	7	1 (0.26, 3.74)
	1-21 days	13	10	1.4 (0.49, 4.42)

# The date of intussusception onset was defined as date of onset of symptoms

## Discussion

An increased risk of intussusception was not detected in any risk window after any dose of the rotavirus vaccine under study in Indian children by either SCCS or case-control analysis. Our post-marketing, active surveillance data provides strong evidence that there is not an adverse safety signal associated with this vaccine in the Indian population.

Our findings differ from post-licensure studies of Rotarix® or RotaTeq® in high- and middle-income countries which found a low-level risk of intussusception after rotavirus vaccination. Studies from Mexico, USA, Australia, England and Singapore have shown a 2.6 to 8.4 fold increase in risk of intussusception in the 21 day period after any dose of Rotarix® vaccination^6-10,28^. Similarly, after RotaTeq® vaccination, Australia and USA have shown a 2.6 to 9 fold increase in risk of intussusception in the 21 day risk period^6,10^. Conversely, our findings appear to be similar to the recent reports from sub-Saharan Africa and South Africa, which did not find an increased risk of intussusception following a different rotavirus vaccine. ^12,13^ (Fig. 3).

There are no defined criteria based on which risk of intussusception in individual children or in populations can be predicted, although the wide variation in background rates of intussusception indicate that there may be population-based predictors^29^. The earlier ages at which rotavirus vaccines are administered in low-income settings (6, 10, and 14 weeks) in contrast to the 2, 4 and 6 months of vaccination in high-income countries may be one reason for this lack of association. Additionally, co-administration of rotavirus vaccine with oral poliovirus vaccine may decrease vaccine rotavirus replication in the intestinal epithelium^30^, thus reducing the likelihood of triggering an intussusception. In Brazil, no increased risk of intussusception was found after the first dose of Rotarix® vaccination, a situation in which this rotavirus vaccine was co-administered with oral polio vaccine^5^. In our study 87.5%, 87.2% and 83% of children received first, second and third doses of rotavirus and oral polio vaccine on the same day, respectively, and no increased risk of intussusception was found after any dose.

The safety findings for two different rotavirus vaccines in Africa and India (the present study) are interesting in the context of reduced vaccine performance in these geographic settings. The immunogenicity and efficacy of oral vaccines, including rotavirus vaccines, are lower in low-resource communities^30,31^. Factors such as inhibition by higher maternal antibodies in serum or breast milk or co-administration of oral polio vaccine that lower the effective titers of vaccine virus, thus reducing vaccine virus replication and hence immunogenicity, might also lower the risk of intussusception. Other factors such as micronutrient deficiencies, malnutrition, environmental enteropathy, and early and constant exposure to other gut pathogens are also proposed to affect mucosal and systemic responses to vaccination^30-32^ and could be responsible for lower background and vaccine associated intussusception rates in low-resource settings.

The present large active surveillance study for intussusception, with high quality countrywide data on intussusception, its management and consequences, including a case-fatality rate, adds safety data to the literature on a relatively new vaccine that is now WHO pre-qualified. Of note, deaths occurred in 1% of Indian infants hospitalized with intussusception whereas in a similar African study, 12% of children with intussusception died^13^.

Our study had certain limitations, which include the exclusion of 12% of eligible children who had inconclusive evidence of vaccination, inability to assess an association with nutrition and the lack of community-based incidence and case-fatality estimates. However, rates of intussusception are not needed for the SCCS analysis as each case acts as his or her own control and was identified independent of its vaccination status. Given the large sample size, the study is adequately-powered to detect small increases in risk in a small window following vaccination and found none. A limitation of the case-control analysis is the relatively smaller size because controls were only enrolled for a subset of cases, and were adjusted for gender, but not for other potential confounders. Nonetheless, risk estimates from both analyses were comparable except for the wider confidence intervals in the case-control analysis.

In summary, the present post-marketing surveillance study indicated that the oral rotavirus vaccine produced in India was not associated with intussusception in the population studied.
