# Supplementary material for: Intussusception after Rotavirus Vaccine Introduction in India
Source: N Engl J Med. Author manuscript; Available in PMC 2020 Nov 12. (PMC7492078; doi:10.1056/NEJMoa2002276)

**Table of contents**


| Sl. no | Content | Page number |
|---|---|---|
| 1. | Figure S1: Flow diagram for cases included in the self-controlled case series analysis to evaluate the association of the rotavirus vaccine under study with intussusception | 2 |
| 2 | Table S1: Site locations and enrolment of intussusception cases in children less than two years of age from April 2016 to June 2019 in Indian states that introduced rotavirus vaccination | 3-4 |
| 3 | Table S2: Brighton Collaboration criteria for intussusception | 5-6 |
| 4 | Table S3: Socio-demographic and clinical characteristics of children included in the self-controlled case series analysis | 7 |
| 5 | Table S4: Comparison of clinical characteristics, treatment modalities and treatment outcomes between vaccinated and unvaccinated children | 8 |
| 6 | Table S5: Follow up of children included in the self controlled case series analysis | 8 |
| 7 | Table S6: Relative incidence of intussusception in the risk periods after first, second and third doses of the rotavirus vaccine under study in infants from 27 hospitals in 10 Indian states by self-controlled case series analysis | 9 |
| 8 | Table S7: Matched odds of intussusception in the risk window after first, second and third dose of rotavirus vaccination in 150 matched case-control pairs taking date of admission as date of onset | 10 |
| 9 | Table S8: Comparison of risk estimates from self-controlled case series analysis and matched case control analysis for 162 intussusception cases included in both analyses | 11 |
| 10 | Figure S2: Flow diagram for cases included in the matched case control analysis | 12 |

Figure S1: Flow diagram for cases included in the self-controlled case series analysis (SCCS) to evaluate the association of the rotavirus vaccine under study with intussusception

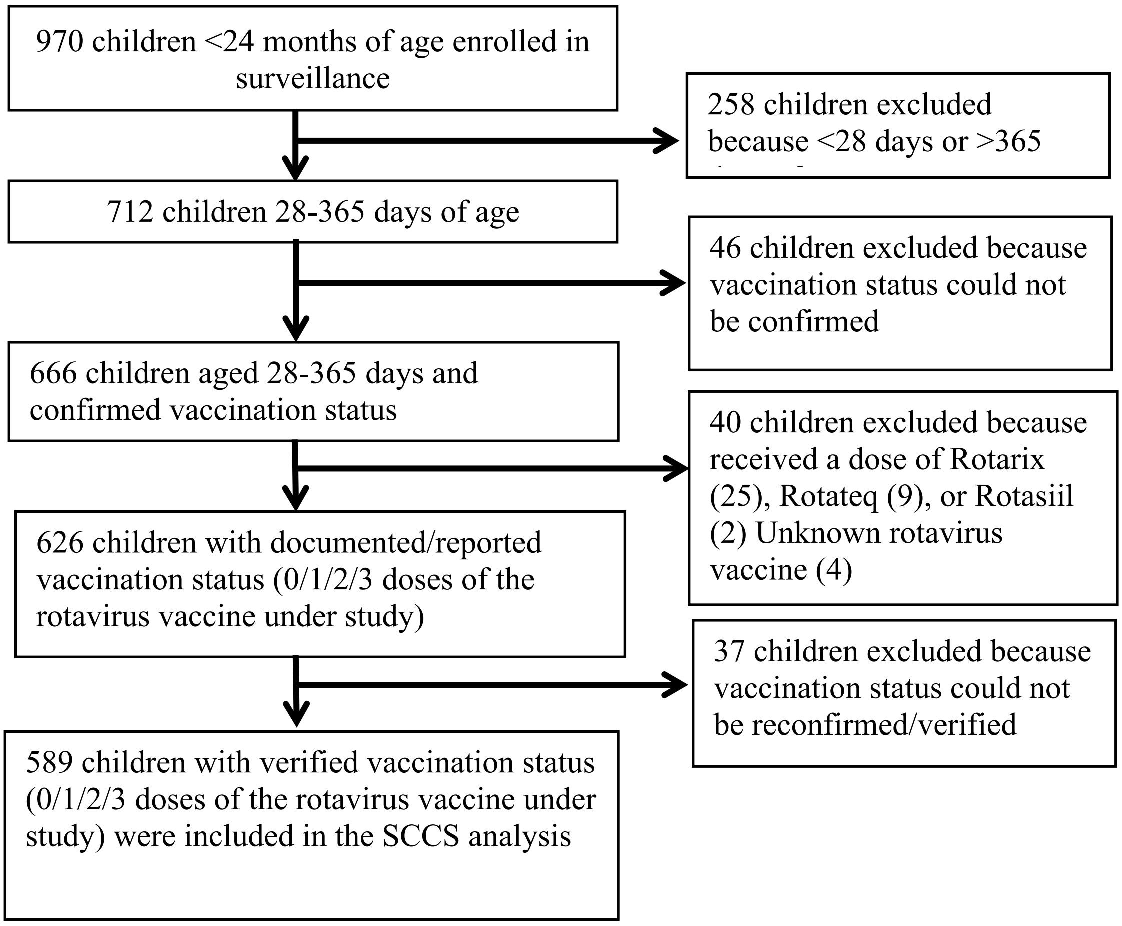

Table S1: Site locations and enrolment of intussusception cases in children less than two years of age from April 2016 to June 2019 in Indian states that introduced rotavirus vaccination

| State | Vaccine introduction date | Hospital name | City | Surveillance period | No. of cases enrolled in surveillance | No. of cases included in the SCCS* analysis |
|---|---|---|---|---|---|---|
| Andhra Pradesh | 20 Apr 2016 | Kurnool Medical College | Kurnool | 01 Jun 2016- 30 Jun 2019 | 22 | 13 |
| | | Government General Hospital and Rangaraya Medical College | Kakinada | 01 Aug 2017- 30 Jun 2019 | 8 | 8 |
| | | King George Hospital and Andhra Medical College | Vishakhapatnam | 01 Jul 2016- 30 Jun 2019 | 12 | 11 |
| | | Sri Venkateshwara Medical College | Tirupati | 01 Jul 2016- 30 Jun 2019 | 20 | 13 |
| Odisha | 26 Mar 2016 | Sardar Valla Bhai Patel Post Graduate Institute of Paediatrics | Cuttack | 15 Apr 2016- 30 Jun 2019 | 80 | 58 |
| | | Kalinga Institute of Medical Sciences | Bhubaneswar | 01 Oct 2016- 30 Jun 2019 | 27 | 14 |
| | | Institute of Medical Sciences and SUM Hospital | Bhubaneswar | 1 Dec 2016- 30 Jun 2019 | 11 | 6 |
| | | Hi-Tech Hospital | Bhubaneswar | 02 Feb 2017- 30 Jun 2019 | 5 | 2 |
| Haryana | 11 Apr 2016 | Pandit Bhagwat Dayal Sharma Post Graduate Institute of Medical Sciences | Rohtak | 02 Jul 2016- 30 Jun 2019 | 21 | 16 |
| | | Shaheed Hasan Khan Mewati Government Medical College | Mewat | 20 Feb 2016- 30 Jun 2019 | 5 | 2 |
| Chandigarh | 11 Apr 2016 | Post Graduate Institute of Medical Education and Research | Chandigarh | 19 Sept 2016- 30 Jun 2019 | 198 | 101 |
| Kerala | No vaccine | Malankara Orthodox Syrian Church Medical College Hospital | Kolencherry | 1 Aug 2016- 30 Jul 2018 | 29 | 18 |
| Tamil Nadu | 20 Sept 2017 | Christian Medical College | Vellore | 20 Sept 2017- 30 Jun 2019 | 36 | 20 |

| | | | | | | |
|---|---|---|---|---|---|---|
| | | Government Vellore Medical College | Vellore | 20 Sept 2017- 30 Jun 2019 | 2 | 1 |
| | | Kanchi Kama Koti Child Trust Hospital | Chennai | 20 May 2017- 30 Jun 2019 | 77 | 26 |
| | | Institute of Child Health | Chennai | 20 Jul 2017- 30 Jun 2019 | 93 | 61 |
| | | Coimbatore Medical College | Coimbatore | 21 Aug 2017- 30 Jun 2019 | 18 | 12 |
| | | Government Rajaji Hospital and Madurai Medical College | Madurai | 26 Dec 2017- 30 Jun 2019 | 23 | 18 |
| Puducherry | No Vaccine | Jawaharlal Nehru Institute of Post-graduate Medical Education & Research | Puducherry | 27 Sept 2017- 30 Jun 2019 | 38 | 26 |
| Rajasthan | 23 Mar 2017 | Sawai Man Singh Medical College | Jaipur | 17 Aug 2017- 30 Jun 2019 | 98 | 73 |
| | | Rabindranath Tagore Medical College | Udaipur | 25 Aug 2017- 30 Jun 2019 | 20 | 12 |
| | | Dr. Sampurnanand Medical College | Jodhpur | 01 Aug 2017- 30 Jun 2019 | 46 | 23 |
| Madhya Pradesh | 03 Apr 2017 | Mahatma Gandhi Memorial Medical College | Indore | 24 Aug 2017- 30 Jun 2019 | 24 | 17 |
| Uttar Pradesh | 16 Jul 2018 | Mangala Hospital & Research Centre | Bijnor | 01 Nov 2018- 30 Jun 2019 | 0 | 0 |
| | 16 Jul 2018 | King George Medical College | Lucknow | 20 Jul 2017- 30 Jun 2019 | 28 | 16 |
| | 16 Jul 2018 | Institute of Medical Sciences, Banaras Hindu University | Varanasi | 21 Apr 2018- 30 Jun 2019 | 10 | 6 |
| Assam | 14 Jun 2017 | Government Medical College | Guwahati | 15 Mar 2018- 30 Jun 2019 | 19 | 16 |
| Total | | | | | 970 | 589 |

*Self-controlled case series

Table S2: Brighton Collaboration Criteria for intussusception

| | |
|---|---|
| Definition | Intussusception is the invagination of one segment of intestine into a segment of distal intestine |
| Level 1 of Diagnostic Certainty | *Surgical criteria*:<br>The demonstration of invagination of the intestine at surgery;<br><br>*and/or Radiologic criteria*:<br>The demonstration of invagination of the intestine by either air or liquid contrast enema; *or*<br>The demonstration of an intra-abdominal mass by abdominal ultrasound with specific characteristic features[2] that is proven to be *reduced* by hydrostatic enema on *postreduction ultrasound*;<br><br>*and/or Autopsy criteria*:<br>The demonstration of invagination of the intestine. |
| Level 2 of Diagnostic Certainty | *Clinical criteria*:<br>Two major criteria (see major and minor criteria for diagnosis below);<br><br>*or*<br>One major criterion[3] and three minor criteria (see major and minor criteria for diagnosis below). |
| Level 3 of Diagnostic Certainty | *Clinical criteria*:<br>Four or more minor criteria (see minor criteria for diagnosis below). |
| Any Level of Diagnostic Certainty | *In the absence of surgical criteria with* the definitive demonstration of an alternative cause of bowel obstruction or intestinal infarction at surgery (e.g., volvulus or congenital pyloric stenosis). |
| Major criteria | 1. *Evidence of intestinal obstruction*:<br>I. History of bile-stained vomiting;<br>*and either*<br>II. Examination findings of acute abdominal distension and abnormal or absent bowel sounds;<br>*Or*<br>III. Plain abdominal radiograph showing fluid levels *and* dilated bowel loops.<br>2. *Features of intestinal invagination*:<br>One or more of the following:<br><br>I. abdominal mass;<br>II. rectal mass;<br>III. intestinal prolapse;<br>IV. plain abdominal radiograph showing a visible intussusceptum or soft tissue mass;<br>V. abdominal ultrasound showing a visible intussusceptum or soft tissue mass; |

| | |
|---|---|
| | VI. abdominal CT scan showing a visible intussusceptum or soft tissue mass.<br>3. *Evidence of intestinal vascular compromise or venous congestion*:<br>I. Passage of blood per rectum;<br>*or*<br>II. Passage of a stool containing “red currant jelly” material;<br>*or*<br>III. Blood detected on rectal examination. |
| Minor criteria | Predisposing factors: age <1 year and male sex;<br><br>• Abdominal pain;<br>• Vomiting;<br>• Lethargy;<br>• Pallor;<br>• Hypovolemic shock;<br>• Plain abdominal radiograph showing an abnormal but non-specific bowel gas pattern. |
| | |

Bines JE, Kohl KS, Forster J, et al. Acute intussusception in infants and children as an adverse event following immunization: case definition and guidelines of data collection, analysis, and presentation. Vaccine 2004;22:569–74.

Table S3: Socio-demographic and clinical characteristics of children included in the self-controlled case series analysis

| Variable | Category | Frequency (%) |
|---|---|---|
| Age | 1-5 months | 219 (37%) |
| | 6-11 months | 370 (63%) |
| Gender | Female | 196 (33%) |
| | Male | 393 (67%) |
| Clinical features | Fever | 202 (34%) |
| | Vomiting | 438 (74%) |
| | Diarrhoea | 240 (41%) |
| | Blood in stools | 481 (82%) |
| | Constipation | 55 (9%) |
| | Abdominal pain | 481 (82%) |
| Location of Intussusception | Ileo-colic | 498 (84%) |
| | Ileo-ileal | 33 (6%) |
| | Colo-colic | 22 (4%) |
| | Compound | 17 (3%) |
| | Unknown | 19 (3%) |
| Treatment modality | Hydrostatic/pneumatic reduction | 200 (34%) |
| | Surgical reduction | 321 (54%) |
| | Intestinal resection | 68 (12%) |
| Treatment outcome | Survived (Discharged home) | 583 (99%) |
| | Died | 6 (1%) |

Table S4: Comparison of clinical characteristics, treatment modalities and treatment outcomes between vaccinated and unvaccinated children

| Variable | Categories | Unvaccinated (N=212) n(%) | Vaccinated (N=377) n(%) | P-value* |
|---|---|---|---|---|
| Fever | Yes | 78 (36.8%) | 124 (32.9%) | 0.33 |
| | No | 134 (63.2%) | 253 (67.1%) | |
| Vomiting | Yes | 162 (76.4%) | 276 (73.2%) | 0.39 |
| | No | 50 (23.6%) | 101 (26.8%) | |
| Diarrhoea | Yes | 80 (37.7%) | 160 (42.4%) | 0.26 |
| | No | 132 (62.3%) | 217 (57.6%) | |
| Constipation | Yes | 27 (12.7%) | 28 (7.4%) | 0.03 |
| | No | 185 (87.3%) | 349 (92.6%) | |
| Abdominal Pain | Yes | 178 (84%) | 303 (80.4%) | 0.28 |
| | No | 34 (16%) | 74 (19.6%) | |
| Blood in stools | Yes | 160 (75.5%) | 321 (85%) | 0.004 |
| | No | 52 (24.5%) | 56 (15%) | |
| Treatment outcome | Death | 1 (0.5%) | 5 (1.3%) | 0.32 |
| | Survived (Discharged home) | 211 (99.5%) | 372 (98.7%) | |
| Intestinal resection | Required | 27 (13%) | 41 (11%) | |
| | Not required | 185 (87%) | 336 (89%) | |
| Repeated episode of Intussusception during follow up# | Yes | 3 (1.8%) | 5 (1.7%) | |
| | No | 166 (98.2%) | 281 (98.5%) | |

**Chi-square test, p-value less than 0.05 was considered statistically significant*
*# 455 children were followed up at 18 months of age*

Table S5: Follow up of children included in the self-controlled case series (SCCS) analysis

| Variable | Frequency |
|---|---|
| No. of children in SCCS analysis | 589 |
| No. of children traced and followed up | 455 |
| Median age at follow up (Inter-quartile range) | 16 (13-22) |
| No. of children who were discharged home and died before reaching the age of follow up | 7 (1.5%) |
| Repeated intussusception episode after current admission | 8 (1.8%) |
| No. of children given rotavirus vaccine after an episode of intussusception | 22 (5%) |

Table S6: Relative incidence (RI, with 95% confidence intervals, CI) of intussusception in the risk periods after first, second and third doses of the rotavirus vaccine under study in infants from 27 hospitals in 10 Indian states by the self-controlled case series analysis#

| Doses of Rotavac vaccine | Risk Period (days) | No. of cases in risk period | RI (95% CI) |
|---|---|---|---|
| Dose 1 | 1-7 days | 2 | 1.02 (0, 4.27) |
| | 8-21 days | 2 | 0.64 (0, 2.05) |
| | 1-21 days | 4 | 0.78 (0.31, 1.96) |
| Dose 2 | 1-7 days | 3 | 0.72 (0.26, 1.94) |
| | 8-21 days | 13 | 1.03 (0.60, 1.76) |
| | 1-21 days | 16 | 0.97 (0.60, 1.57) |
| Dose 3 | 1-7 days | 14 | 1.40 (0.81, 2.42) |
| | 8-21 days | 20 | 0.95 (0.60, 1.49) |
| | 1-21 days | 34 | 1.09 (0.76, 1.57) |

# The date of intussusception onset was defined as the date of admission at the surveillance hospital

Table S7: Matched odds of intussusception in the risk window after first, second and third dose of rotavirus vaccination in 150 matched case-control pairs#

| Doses of rotavirus vaccine under study | Risk window relative to reference date | No. of cases in risk window | No. of controls in risk window | Matched odds ratio |
|---|---|---|---|---|
| Dose 1 | 1-7 days | 1 | 0 | 0 |
| | 8-21 days | 1 | 5 | 0 (0, 1.51) |
| | 1-21 days | 2 | 5 | 0 (0, 2.42) |
| Dose 2 | 1-7 days | 1 | 0 | 0 |
| | 8-21 days | 2 | 4 | 0.33 (0.01, 4.15) |
| | 1-21 days | 3 | 4 | 0.66 (0.05, 5.81) |
| Dose 3 | 1-7 days | 6 | 2 | 5 (0.56, 236.48) |
| | 8-21 days | 5 | 6 | 0.8 (0.16, 3.72) |
| | 1-21 days | 11 | 8 | 1.6 (0.46, 6.22) |

# Sensitivity analysis using the date of onset of intussusception as the date of admission at the surveillance hospital.

Table S8: Comparison of risk estimates from self-controlled case series analysis (SCCS) and matched case control analysis[@] for 162 intussusception cases included in both analyses[#]

| Doses of rotavirus vaccine under study | Risk Period# (days) | No. of cases in risk period | SCCS analysis RI (95% CI) | Case-control analysis Matched Odds Ratio (95% CI) |
|---|---|---|---|---|
| Dose 1 | 1-7 days | 1 | 0.89 (0-5.37) | 1 (0.12-78.49) |
| | 8-21 days | 1 | 0.45 (0-2.09) | 0 (0-1.51) |
| | 1-21 days | 2 | 0.63 (0-2.39) | 0 (0-1.51) |
| Dose 2 | 1-7 days | 1 | 0.54 (0-2.45) | 1 (0.01-78.49) |
| | 8-21 days | 3 | 0.66 (0-2.08) | 1 (0.07-13.79) |
| | 1-21 days | 4 | 0.61 (0.12-1.76) | 1 (0.13-7.46) |
| Dose 3* | 1-7 days | 6 | 2.30 (0.68-5.53) | 2.5 (0.41-26.25) |
| | 8-21 days | 7 | 1.28 (0.42-2.76) | 1 (0.26-3.74) |
| | 1-21 days | 13 | 1.57 (0.73-3.04) | 1.4 (0.49-4.42) |

# The date of onset of intussusception was defined as the date of symptom onset

[@]In the case-control analysis to detect an odds ratio of 2, with data from our study showing 66% of controls vaccinated with at least one dose, with one control enrolled per each case, at 5% level of significance, with 162 case control pairs, the power of the study is 78%.

*For dose 3, the 1-7 day window, although not significant, the RI for the SCCS is 2.3 and the matched odds ratio for the case-control analysis is 2.5. It is feasible that that there may be a difference in risk from dose 3 (or any dose) depending on whether the dose was given on time or later when intussusception peaks, but the study is not powered to examine age related differences.

Figure S2: Flow diagram for cases included in the matched case control analysis

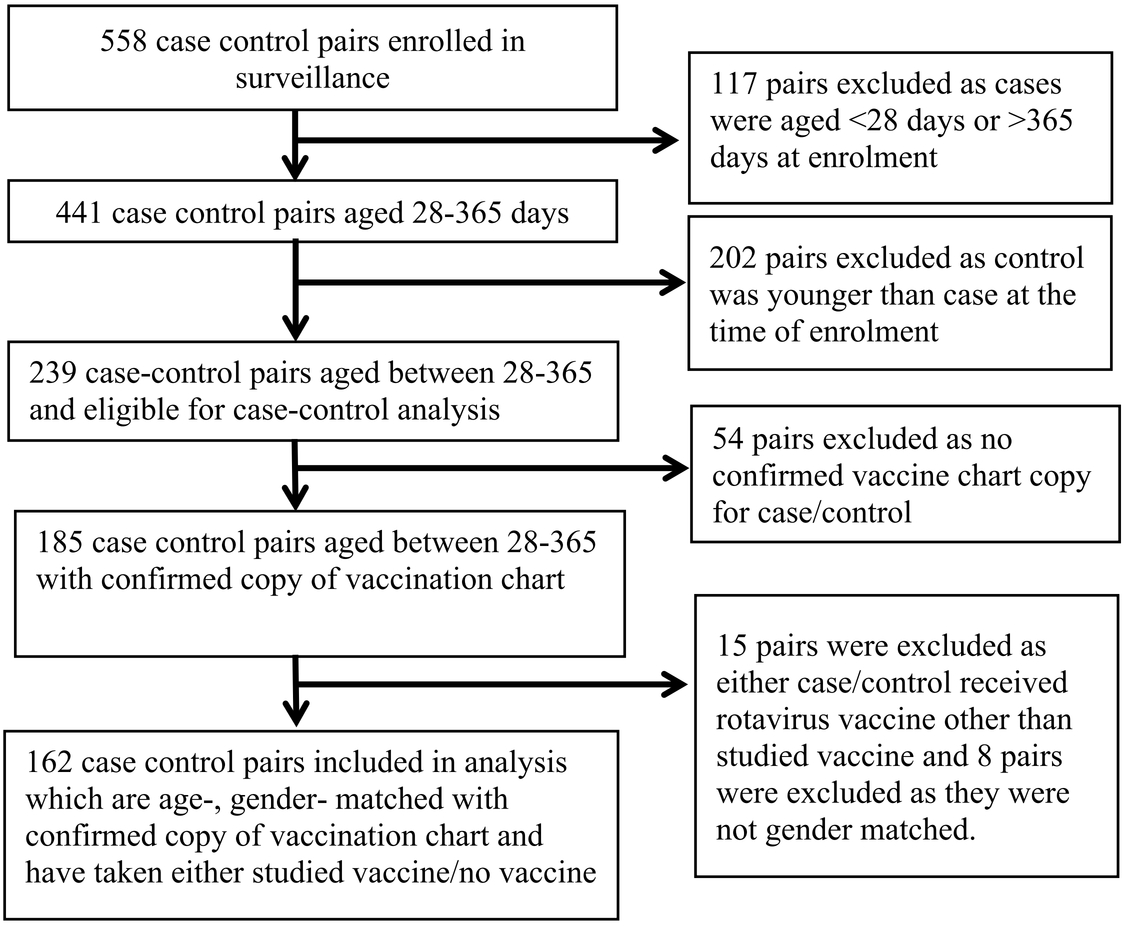

Supplement: Supplementary file 1 [file NEJM-2020-2002276-s1.pdf]
